# On the Viability of Diffusion MRI-Based Microstructural Biomarkers in Ischemic Stroke

**DOI:** 10.3389/fnins.2018.00092

**Published:** 2018-02-21

**Authors:** Ilaria Boscolo Galazzo, Lorenza Brusini, Silvia Obertino, Mauro Zucchelli, Cristina Granziera, Gloria Menegaz

**Affiliations:** ^1^Department of Computer Science, University of Verona, Verona, Italy; ^2^Translational Imaging in Neurology Group, Department of Neurology, Basel University Hospital, Basel, Switzerland

**Keywords:** diffusion propagator, tensor model, 3D-SHORE model, reproducibility, tract-based, gray matter, ischemic stroke

## Abstract

Recent tract-based analyses provided evidence for the exploitability of 3D-SHORE microstructural descriptors derived from diffusion MRI (dMRI) in revealing white matter (WM) plasticity. In this work, we focused on the main open issues left: (1) the comparative analysis with respect to classical tensor-derived indices, i.e., Fractional Anisotropy (FA) and Mean Diffusivity (MD); and (2) the ability to detect plasticity processes in gray matter (GM). Although signal modeling in GM is still largely unexplored, we investigated their sensibility to stroke-induced microstructural modifications occurring in the contralateral hemisphere. A more complete picture could provide hints for investigating the interplay of GM and WM modulations. Ten stroke patients and ten age/gender-matched healthy controls were enrolled in the study and underwent diffusion spectrum imaging (DSI). Acquisitions at three and two time points (*tp*) were performed on patients and controls, respectively. For all subjects and acquisitions, FA and MD were computed along with 3D-SHORE-based indices [Generalized Fractional Anisotropy (GFA), Propagator Anisotropy (PA), Return To the Axis Probability (RTAP), Return To the Plane Probability (RTPP), and Mean Square Displacement (MSD)]. Tract-based analysis involving the cortical, subcortical and transcallosal motor networks and region-based analysis in GM were successively performed, focusing on the contralateral hemisphere to the stroke. Reproducibility of all the indices on both WM and GM was quantitatively proved on controls. For tract-based, longitudinal group analyses revealed the highest significant differences across the subcortical and transcallosal networks for all the indices. The optimal regression model for predicting the clinical motor outcome at *tp3* included GFA, PA, RTPP, and MSD in the subcortical network in combination with the main clinical information at baseline. Region-based analysis in the contralateral GM highlighted the ability of anisotropy indices in discriminating between groups mainly at *tp1*, while diffusivity indices appeared to be altered at *tp2*. 3D-SHORE indices proved to be suitable in probing plasticity in both WM and GM, further confirming their viability as a novel family of biomarkers in ischemic stroke in WM and revealing their potential exploitability in GM. Their combination with tensor-derived indices can provide more detailed insights of the different tissue modulations related to stroke pathology.

## Introduction

In the last 30 years, diffusion magnetic resonance imaging (dMRI) has been proven to be a valuable tool for characterizing physiological and pathological conditions *in-vivo* (Le Bihan et al., 1986; Beaulieu, 2002). An increasing number of modeling methods have been proposed for inferring tissue microstructural properties from the acquired diffusion signal (for a detailed overview see Novikov et al., 2016), many of which rely only on the reconstruction of the ensemble average propagator (EAP), i.e., the probability distribution function of the water molecules displacements. The EAP, under some optimality assumptions, contains the full information about the diffusion process and therefore can inform about the underlying tissue architecture (Zucchelli et al., 2016b), leading to numerical indices that can indirectly quantify the different microstructural features.

Diffusion Tensor Imaging (DTI) (Basser et al., 1994a) was the first EAP model introduced to describe the anisotropic nature of the diffusion process in biological tissues and is still the preferred method in clinical settings thanks to its ability to estimate the principal diffusion direction from very few dMRI measurements. The scalar indices obtained from DTI, mainly the mean diffusivity (MD) and the fractional anisotropy (FA) (Pierpaoli and Basser, 1996), have become precious tools for characterizing pathological conditions such as tumors, stroke and neurodegenerative disorders (Sundgren et al., 2004). Nonetheless, DTI has an inherent strong modeling constraint related to the description of the EAP as a single multivariate Gaussian function. This assumption is rarely adequate in real conditions where complex white matter (WM) topologies featuring crossing, fanning and kissing fibers are most often encountered, severely limiting its applicability. Among the widespread EAP models proposed for circumventing this limitation, one of the most accurate is the Simple Harmonic Oscillator Based Reconstruction and Estimation (SHORE), firstly introduced in Özarslan et al. (2008). 3D-SHORE and its extensions, as Mean Apparent Propagator (MAP)-MRI (Özarslan et al., 2013), demonstrated good performance in detecting multiple diffusion directions and are among the most promising EAP-based models for characterizing the tissue microstructure, as recently highlighted at the SPARC-dMRI contest (Ning et al., 2015). Under some assumptions, reliable measures of tissue anisotropy can be derived from these EAP models, such as the Generalized Fractional Anisotropy (GFA) and the Propagator Anisotropy (PA), along with measures of the EAP variance (Mean Square Displacement, MSD). In addition, they provide indices that quantify various features of the three-dimensional diffusion process, namely the Return to the Origin Probability (RTOP), the Return To the Axis Probability (RTAP) and the Return To the Plane Probability (RTPP). When the diffusion time is long enough and under narrow pulse assumptions (Özarslan et al., 2013), these indices reflect the degree of restriction of the water molecules in the voxel, which is linked to the underlying pore shape and thus represent relevant descriptors of the microstructural properties (Zucchelli et al., 2016a).

Since their first introduction, 3D-SHORE indices have been increasingly explored as novel potential biomarkers of brain microstructure. This has been shown both on synthetic data and in *ex-vivo* experiments on a marmoset brain (Özarslan et al., 2013) as well as in *in-vivo* studies on healthy subjects (Avram et al., 2014; Fick et al., 2015; Mendez et al., 2016; Zucchelli et al., 2016a). Very few studies have tried to pursue their potentialities as clinical biomarkers in pathologies, with promising results to date only on Alzheimer's animal models (Fick et al., 2016) and on ischemic stroke (Brusini et al., 2015; Obertino et al., 2016). In the latter case, albeit DTI scalar indices have been used to assess stroke features in several longitudinal studies (Maniega et al., 2004; Yu et al., 2009), the characterisation of the network pathophysiology with advanced EAP-based indices would add insights into the reorganization processes that can be combined with clinical information to draw a more precise picture of the disease. A recent study (Brusini et al., 2016) investigated these aspects on a group of ischemic stroke patients and assessed the performance of selected 3D-SHORE indices along WM tracts of different motor networks (cortical, subcortical, and transcallosal circuits). Results highlighted how 3D-SHORE-based indices (mainly GFA, PA, RTAP, and RTPP) could provide measurements featuring high precision and allow discriminating patients from controls, supporting their suitability for mapping longitudinal changes after stroke.

Although the available findings for these numerical indices are encouraging, a quantitative comparison with the classical tensor-derived metrics is currently lacking but essential to further probing their potentialities as biologically specific markers. Indeed, MD and FA remain the standard measures in clinical settings, especially for acute stroke imaging. Therefore, 3D-SHORE-based indices have to be carefully related to tensor-derived indices in terms of precision, consistency, discriminative and predictive power in patients, all essential requirements to be eligible as numerical biomarkers. Avram et al. (2016) reported a first attempt to assess the feasibility of novel EAP-indices (from MAP-MRI modeling rather than 3D-SHORE) in comparison to classical DTI indices, demonstrating good consistency across subjects and reproducibility in test–retest experiments on three controls. However, despite the promising results, the authors dealt with a very limited number of healthy subjects and relied only on qualitative visual comparisons, acknowledging the need for further studies on patient populations that, to the best of our knowledge, are still missing in recent literature.

Whereas a great research effort has been devoted to dMRI signal modeling in WM, its exploitability for characterizing gray matter (GM) structures is still largely unexplored. In fact, there is a growing need for a more comprehensive assessment of GM tissue changes using dMRI. The intrinsic complexity of GM microstructure which, as opposed to WM, lacks coherent tissue orientation complicates the modeling and interpretation of the diffusion process, and casts shadows on the suitability of the currently available models. Some previous studies with classical DTI indices have highlighted MD as a promising marker of GM diffusivity changes in several pathologies such as Alzheimer's disease (Weston et al., 2015), multiple sclerosis (Ceccarelli et al., 2007), and Parkinson (Kim et al., 2013). However, DTI is scarcely employed in the assessment of GM regions, especially in the cortex, and its ability of capturing microstructural features and feature modulations in GM is still under debate. Conversely, thanks to the ability of capturing the EAP in complex tissue microstructures, the 3D-SHORE model might allow characterizing the signatures of hindered diffusion in GM regions as well as providing information about GM changes occurring over time.

The goal of this study was twofold. First, to complete the assessment of the potential of the 3D-SHORE-derived indices in capturing the microstructural feature modulations induced by ischemic stroke in WM by providing a comparative analysis of their performance with respect to the classical DTI-based FA and MD indices. Second, to start bridging WM and GM modeling by investigating the ability of the considered models (DTI and 3D-SHORE) for the identification of microstructural feature variations in GM, possibly hinting at plasticity processes.

## Materials and methods

### Dataset

Ten ischemic stroke patients (6 males, mean age: 60.3 ± 12.3 years) and ten age- and gender-matched healthy subjects were enrolled in the study and underwent longitudinal MRI acquisitions on a 3T Siemens scanner (Trio, Siemens, Erlangen, Germany), as firstly reported in Granziera et al. (2012b). Of note, an optimized protocol and a dedicated 32-channel head coil with excellent signal-to-noise (SNR) properties (based on Wiggins et al., 2006) were employed, aiming at maximizing the SNR in the acquired data (as in Granziera et al., 2009). Acquisitions were performed at three time points in patients (within 1 week (*tp1*), 1 month (± 1 week, *tp2*), and 6 months (± 15 days, *tp3*) after the injury), and at two time points in controls (1 month apart, *tp1c*, and *tp2c*). The same structural imaging protocol was used in all cases. In particular, Diffusion Spectrum Imaging (DSI), a high angular resolution diffusion technique (Wedeen et al., 2005), was performed using a single-shot spin-echo echo-planar imaging (EPI) product sequence and the following parameters: TR/TE = 6,600/138 ms, FOV = 212 × 212 mm^2^, 34 slices, 2.2 × 2.2 × 3 mm^3^ resolution, GRAPPA = 2, scan time = 25.8 min. The sampling scheme consisted of a keyhole Cartesian acquisition with 258 diffusion directions covering a half q-space 3D grid with radial grid size of 5. Thirty-four different *b*-values (from 300 up to 8,000 s/mm^2^) were included in the acquisition and one image was acquired at *b* = 0 s/mm^2^ (b0 volume). Because of the inherent antipodal symmetry, the signal was duplicated on the other hemisphere yielding to 515 points.

In order to provide a measure of the diffusion data quality, SNR values were calculated for all the b0 volumes as the ratio of the mean of the signal divided by the standard deviation of the underlying Gaussian noise (Descoteaux et al., 2011). A uniform ROI in the background was chosen for deriving the noise standard deviation while the mean signal was extracted from the corpus callosum, selected as representative ROI for the SNR calculation. The estimated values are reported in Table 1. High-resolution 3D T1-weighted images were also added to the protocol (TR/TE = 2,300/3 ms, FOV = 256 × 256 mm^2^, 160 slices, 1 × 1 × 1.2 mm^3^ resolution, scan time = 6.13 min). Besides MRI acquisitions, patients underwent clinical neurological assessment following the National Institutes of Health Stroke Scale (NIHSS) at each *tp*. Only the motor part of the NIHSS score was retained for further analysis. Stroke volumes were derived from the individual high-resolution T1-weighted images using the statistical parametric mapping (SPM) lesion segmentation toolbox (www.fil.ion.ucl.ac.uk/spm/). All the subjects signed the written informed consent to the imaging in accordance with the Declaration of Helsinki and the Lausanne University Hospital approved the protocol. Patient demographics and main clinical information are reported in Supplementary Table 1.

**Table 1:** Signal-to-Noise (SNR) ratio for the diffusion datasets.

**SNR-corpus callosum**		
Controls	*tp1*	28.47 ± 5.33
	*tp2*	28.63 ± 4.38
Patients	*tp1*	28.21 ± 4.60
	*tp2*	29.65 ± 6.24
	*tp3*	27.25 ± 4.55

*SNR values were calculated on the b0 volume of each subject. In particular, a uniform ROI in the background was chosen for estimating the noise standard deviation while the mean signal was extracted from the corpus callosum, selected as representative ROI for the SNR calculation. Mean ± standard deviation values across subjects are reported, considering each time point and group separately*.

### Signal modeling and microstructural descriptors

The classical DTI (Basser et al., 1994a,b) and the 3D-SHORE (Özarslan et al., 2008, 2013) models were used to reconstruct the EAP from which the microstructural descriptors were then derived.

The EAP can be recovered from the diffusion weighted signal attenuation *E*(*q*) under the narrow pulse assumption (Stejskal and Tanner, 1965) via the Fourier relationship:

(1) $$P\left(r\right)=\int_{q\in R^{3}}E\left(q\right)e^{i2\pi qr}dq$$

where *P*(***r***) is the EAP, indicating the likelihood for a particle to undergo a net displacement ***r*** in the unit time and ***q*** = *q**u*** is the sampling position, with ***u*** being unit vector of the reciprocal space, or *q*-space.

DTI assumes that the diffusion propagator can be described by a single 3D Gaussian distribution (Basser et al., 1994a,b) from which a 3 × 3 symmetric positive-definite matrix is derived (*D*, diffusion tensor) and used to compute the classical tensor-based indices (MD and FA) as follows:

(2) $$MD=\frac{\left(\lambda_{1}+\lambda_{2}+\lambda_{3}\right)}{3}$$

(3) $$FA=\sqrt{\frac{1}{2}\frac{{\left(\lambda_{1}-\lambda_{2}\right)}^{2}+{\left(\lambda_{2}-\lambda_{3}\right)}^{2}+{\left(\lambda_{1}-\lambda_{3}\right)}^{2}}{\lambda_{1}^{2}+\lambda_{2}^{2}+\lambda_{3}^{2}}}$$

where λ_1_, λ_2_, λ_3_ are the eigenvalues of *D*. Only *b* < 1,500 mm^2^/s were used for the DTI analysis, corresponding to 32 gradient directions.

The novel microstructural indices explored in this work were calculated by fitting the SHORE model (Özarslan et al., 2008, 2013) based on the solutions of the 3D quantum harmonic oscillator in the formulation using the orthonormalized basis:

(4) $$E\left(q\right)=\sum_{l=0,even}^{N_{max}}\sum_{n=l}^{\frac{\left(N_{max}+l\right)}{2}}\sum_{m=-l}^{l}c_{nlm}\Phi_{nlm}\left(q\right)$$

In this equation, *N*_*max*_ is the maximal order of the functions, Φ_*nlm*_(***q***) are the functions forming the 3D-SHORE orthonormal basis and are given by:

(5) $$\begin{array}{l} \Phi_{nlm}\left(q\right)={\left[\frac{2\left(n-l\right)!}{\zeta^{\frac{3}{2}}\Gamma \left(n+\frac{3}{2}\right)}\right]}^{\frac{1}{2}}{\left(\frac{q^{2}}{\zeta }\right)}^{\frac{l}{2}}\\ \;exp\left(\frac{-q^{2}}{2\zeta }\right)L_{n-l}^{l+\frac{1}{2}}\left(\frac{q^{2}}{\zeta }\right)Y_{l}^{m}\left(u\right) \end{array}$$

where Γ is the Gamma function and ζ is a scaling parameter determined by the diffusion time and the mean diffusivity (Merlet and Deriche, 2013; Zucchelli et al., 2016a). For the 3D-SHORE model, the EAP is obtained by plugging Equation (4) into Equation (1) (Özarslan et al., 2013; Zucchelli et al., 2016a). Due to the linearity of the Fourier transform, the EAP basis is thus expressed in terms of the same set of coefficients *c*_*nlm*_ as the diffusion signal.

RTAP and RTPP (Özarslan et al., 2013) represent the integral of the EAP along the main diffusion direction and over the plane passing through the origin and perpendicular to the main diffusion direction, respectively:

(6) $$RTAP=\int_{R}P\left(r_{∥}\right)dr_{∥}$$

(7) $$RTPP=\int_{R}P\left(r_{⊥}\right)d^{2}r_{⊥}$$

where ***r***_∥_ is the main diffusion direction, and ***r***_⊥_ indicates the plane orthogonal to the main diffusion direction and passing through the origin. It has been shown (Özarslan et al., 2013; Zucchelli et al., 2016b) that, under the assumptions of narrow pulses and long diffusion time, RTAP and RTPP are proportional to the inverse of the mean apparent cross-sectional area and length of the compartment where diffusion takes place, respectively.

The MSD represents the mean square displacement of the water molecules in the unit time and is computed as follows:

(8) $$MSD=\int_{R^{3}}P\left(r\right)r^{2}d^{3}r$$

MSD has been proven to be closely related to the classical MD index, sharing similar patterns (Wu and Alexander, 2007).

From the EAP it is possible to derive a propagator anisotropy index, depending on the angular distance between the isotropic part of the EAP, that is encoded in the coefficients *c*_*n*00_, and the full EAP as in Özarslan et al. (2013):

(9) $$PA\;=\sqrt{1-\frac{\sum_{n=0}^{N_{\max}}c_{n00}^{2}}{\sum_{n,l,m}^{N_{\max}}c_{nlm}^{2}}}$$

Finally, the Orientation Distribution Function (ODF) can be analytically obtained from the 3D-SHORE by taking the radial integral of the EAP along a given direction (Merlet and Deriche, 2013; Özarslan et al., 2013). From the ODF it is possible to derive another measure of anisotropy, the GFA index, which can be viewed as the normalized variance of the ODF:

(10) $$GFA=\sqrt{\frac{n\sum_{i=1}^{n}{\left(ODF\left(u_{i}\right)-\langle ODF\rangle \right)}^{2}}{\left(n-1\right)\sum_{i=1}^{n}ODF{\left(u_{i}\right)}^{2}}}$$

where *ODF*(***u***_*i*_) is the value of the ODF in the direction ***u***_*i*_, and 〈*ODF*〉 is the mean ODF value across all directions.

In this work, we used both classical tensor-based indices (MD, FA) along with the aforementioned 3D-SHORE-based indices (RTAP, RTPP, MSD, PA, and GFA) to detect microstructural modulations by both tract-based analyses in WM and by ROI-based analyses in GM, respectively. While the first allowed assessing the performance of the 3D-SHORE-based indices with respect to FA and MD in the motor cortical and subcortical networks, the second targets the GM in order to provide a more complete picture of changes occurring after stroke and possibly pointing at plasticity processes.

### Tract-based analysis of WM

The tractogram was obtained via a streamline-based algorithm with diffusion tensor ODFs computed from the DSI images (Diffusion Toolkit, CMTK, www.connectomics.org). Individual high-resolution T1-weighted images were parcellated using Freesurfer (http://surfer.nmr.mgh.harvard.edu/) and the Desikan-Killiany anatomical atlas at 83-region scale (sixty-four cortical and nineteen subcortical regions) plus the corpus callosum was employed. The FLIRT tool from the FMRIB FSL software (www.fmrib.ox.ac.uk/fsl) was used for the linear (affine) registration of the T1-weighted scan to diffusion data. In particular, the diffusion baseline images (b0 volumes) were considered as reference images for estimating the registration transformation subsequently applied to back-project the subject-specific anatomical parcellation into the DSI space.

**Figure 1:**
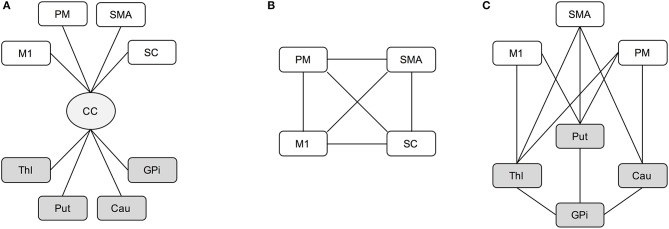
Schematic representation of transcallosal **(A)**, cortical **(B)**, and subcortical **(C)** networks. Cortical areas (white): M1, primary motor area; SMA, supplementary motor area; SC, somatosensory cortex; PM, premotor area. Subcortical nuclei (gray): Thl, thalamus; Cau, caudatus; Put, putamen; GPi, globus pallidus. Bundle of neural fibers (light gray): CC, corpus callosum.

Among all the parcels, a subset of the motor regions of interest (ROIs) was considered for the analyses. For the cortical area we selected the primary motor area (M1), supplementary motor area (SMA), somatosensory cortex (SC) and premotor area (PM), which was considered as a unique region given by the joint combination of the dorsal and ventral parts from the Freesurfer parcellation, while thalamus (Thl), caudatus (Cau), putamen (Put), and globus pallidus (GPi) were selected for the subcortical part. Then, three loops involved in the motor network and linking these cortical-subcortical ROIs were considered in the analysis as in Brusini et al. (2016). In details, the transcallosal circuit (CC) gathers the set of fibers linking the corpus callosum with each considered ROI (Figure 1A). The cortical loop (CORT) consists of fibers linking the four cortical ROIs (Figure 1B), while the subcortical loop (SUBCORT) includes the set of fibers linking cortical (except SC) with subcortical ROIs (Figure 1C).

Tensor-based and 3D-SHORE-based indices were finally calculated along each fiber bundle linking every pair of regions in the proposed networks. To this end, the values of the considered microstructural parameter were firstly mapped onto each fiber connecting two specific ROIs, then averaged across the whole fiber bundle. In this way, a representative microstructural value was derived for each connection of the considered network.

### Region-based analysis of GM

The individual high-resolution T1-weighted images were segmented into WM, GM, and cerebrospinal fluid (CSF) tissues using the SPM toolbox (Friston et al., 1995). A binary mask was derived for GM using a conservative 95% threshold on the individual probability maps.

Eighty regions from the Freesurfer parcellation were considered (brainstem and corpus callosum were excluded) and masked with the binary GM mask. Four small subcortical regions per hemisphere resulted to be empty after GM masking and were excluded from further analyses, for a total of seventy-two regions. For all indices, the mean GM value across each masked ROI was then calculated. In particular, average measures were calculated across corresponding regions in both hemispheres for controls, while averaging was constrained to the contralateral hemisphere for patients, leading in both cases to thirty-six representative GM values for each index and subject. The list of the considered regions and relative abbreviations is provided in Supplementary Table 2.

### Test–retest reproducibility analysis

Before comparing the performance of the indices in the two groups and assessing their discriminative/predictive power, a preliminary step for analyzing their variability and longitudinal stability was performed following the test-retest paradigm on controls (*tp1c* and *tp2c*). This allowed to quantitatively assess their reproducibility in physiological conditions and thus to estimate the precision of the measurements. These elements were quantified for all the microstructural indices, relying on all the representative measures coming from both tract-based and region-based analysis.

The following metrics were computed for each measure to assess the reproducibility: the intraclass correlation coefficients (ICC) and the intra- and inter-subject coefficients of variation (CV_intra_ and CV_inter_) (Bland and Altman, 1996; Chen et al., 2011; Pinto et al., 2016). ICC is one of the most important methods to assess the reliability of a measure, reflecting both intra- and inter-subject variability. It allows evaluating how measurements derived from the same subject are reproducible across sessions, taking into account the intra/inter-subject variability as follows:

(11) $$ICC=\frac{\sigma_{bs}^{2}}{\sigma_{bs}^{2}+\sigma_{ws}^{2}}$$

where σ_*bs*_ is the between-subject standard deviation and σ_*ws*_ is the within-subject standard deviation for repeated measurements. ICC levels and reliability can be evaluated using the following recommendations: poor (< 0.4), fair (0.41–0.59), good (0.60–0.74) and excellent (>0.75) (Fleiss, 1981; Cicchetti, 2010).

The CV_intra_ (within-subject CV) measures the variability between sessions of the same subject, reflecting both physiological variations that can occur in a natural way and possible measurement errors (Pinto et al., 2016). CV_intra_ was computed as:

(12) $$CV_{intra}=\frac{\sigma_{ws}}{\mu }\cdot 100\left[\%\right]$$

where μ is the mean value of the parameter across subjects and sessions (overall mean). Since only two measurements per subject were available, σ_*ws*_ can be calculated as:

(13) $$\sigma_{ws}=\sqrt{\left(\frac{\sum_{i=1}^{k}{\left(a_{itp1}-a_{itp2}\right)}^{2}}{2\times k}\right)}$$

where *k* is the number of subjects, and *a*_*itp*1_and *a*_*itp*2_ are the measurements for subject *i* on test (*tp1*) and retest (*tp2*) sessions, respectively (Bland and Altman, 1996).

Finally, the CV_inter_ (between-subject CV) measures the stability across the group, reflecting the inter-individual variability. For each index, the CV_inter_ was initially computed for each session as follows:

(14) $$CV_{inter_{j}}=\frac{\sigma_{tpj}}{\mu_{tpj}}\cdot 100\left[\%\right]$$

where *tpj* represents the session *j* (*j* = 1, 2), μ_*tpj*_ and σ_*tpj*_ are the mean and standard deviation values, respectively, calculated across all the subjects for the considered session *tpj*. The representative CV_inter_ measure was then computed as the mean of the CV_interj_ from the two sessions.

For biological measurements from MRI, CV_intra_ ≤ 10% and CV_inter_ < 15% are considered as acceptable (Heiervang et al., 2006; Marenco et al., 2006).

For tract-based measures, ICC and CV_intra_ were a single measure for each loop, as all the connections belonging to the corresponding network were grouped for providing a global representative measure of network reproducibility, in line with (Brusini et al., 2016). Conversely, the representative CV_inter_ metric was first computed for each tract and then summarized for each loop by the mean ± standard deviation (SD) values across connections. This allowed to evaluate the stability across subjects and also the inter-subject variability across the different structural links of each network.

For region-based analysis, CV_intra_ and ICC were computed for each ROI individually (mean ± SD values across GM ROIs), while the representative CV_inter_ metric was initially calculated for each region and then reported as mean ± SD values across GM ROIs. This again allowed to appreciate the variability across the GM structures.

### Statistical analysis on tract-based outcomes–patients and controls

After the reproducibility analysis, the outcome measures from tract-based analysis were assessed for depicting possible differences between patients and controls and determining the discriminative power of the different indices. In particular, for each index and network, the percentage absolute changes in mean values between *tp* (Δ_*tp*_) were calculated as in Brusini et al. (2016).

Since the Kolmogorov–Smirnov normality test confirmed the normal distribution of the percentage values, statistical comparisons with the unpaired *t*-test were performed to detect significant differences between delta changes in controls (Δ_*tp*12*c*_) and Δ_*tp*12_, Δ_*tp*23_, Δ_*tp*13_ calculated in the patient cohort. While in our previous work (Brusini et al., 2016) the False Discovery Rate (FDR) correction was applied to the statistical results, here a more conservative Bonferroni adjustment (α = 0.05) was used to correct for multiple comparisons across indices. This approach was chosen in order to further strengthen the statistical findings and highly reduce false positive results.

In addition, in order to assess the predictive power of both tensor-derived and 3D-SHORE-derived indices, different linear regression models were considered and their performance in predicting the clinical motor outcome at 6 months (NIHSS at *tp3*) was tested. First, a linear regression model including only clinical information at baseline (age, stroke size, and NIHSS motor scores at *tp1*) as predictors was calculated for reference. Then, for each network, three types of regression models were built and compared as opposed to what was done in our previous work (Brusini et al., 2016), where a single model combining clinical information with a set of 3D-SHORE-based descriptors (GFA, PA, R, RTAP, RTOP, RTPP) was considered. In detail, the following models were considered:

1. *Tensor-based model (TBM)*: the average across all the connections of the considered loop at *tp1* was calculated for each index (MD, FA) and both mean values were included as predictors along with age, stroke size and NIHSS at *tp1*.
2. *3D-SHORE-based model (SBM)*: the average across all the connections of the considered loop at *tp1* was calculated for each index (GFA, PA, RTAP, RTPP, MSD) and these mean values were included as predictors along with age, stroke size and NIHSS at *tp1*.
3. *Global microstructural model (GBM)*: all the indices at *tp1* (both tensor-derived and 3D-SHORE-derived) were included as predictors, after having calculated their individual mean value across all the connections of the considered loop. No clinical information was included.

All the linear regression analyses were performed in SPSS, version 18 (SPSS, Inc., Chicago, Illinois), setting *p* = 0.05 as significance threshold of the overall *F*-test to determine whether the regression model significantly predicts the clinical motor outcome. A backward elimination strategy was utilized to obtain a parsimonious regression model. In details, a full model that includes all the predictor variables was initially created. Then, each subsequent step removed the least significant variable in the model until all the remaining variables had individual *p*-values smaller than the selected criterion. The default criterion in SPSS (based on the probability of F-to-remove, with *pout* = 0.10) was chosen for deleting a predictor that had little or no influence on the dependent variable. For each optimal model, the calculated *R*^2^ value was adjusted for the number of predictors included, in order to perform a valid comparison across the different regression models and penalize the addition of extraneous predictors. The following equation, as implemented in SPSS (Ezekiel, 1930; Kirk, 1996), was applied:

(15) $$R_{adj}^{2}=1-\frac{\left(1-R^{2})(N-1\right)}{N-k-1}$$

where *N* is the sample size and *k* is the number of predictors in the corresponding model, i.e., those that were not deleted by the backward selection process, excluding the constant.

### Statistical analysis on GM region-based outcomes–patients and controls

In order to compare the GM region-based measures, a three-way mixed (within-between) analysis of variance (ANOVA) was firstly performed for each microstructural index to test the significance of different factors, using the mean index value as dependent variable. Three independent variables were considered: Time with two levels and Region with thirty-six levels (within-subject factors) plus Group with two levels as between-subject factor. In addition, a further two-way repeated measures ANOVA was performed on the patient group data in order to assess for the presence of longitudinal changes in contralateral GM structures across all temporal scales. Also in this case the mean value for each index was used as dependent variable in the corresponding ANOVA, while two independent variables were included: Time with three levels and Region with thirty-six levels.

For each ANOVA, Mauchley test was used to assess the sphericity assumption and Greenhouse-Geisser epsilon adjustments for non-sphericity were applied where appropriate. *Post-hoc* tests adjusted for multiple comparisons with the Bonferroni correction were used when significant interactions were found. For all statistical tests, performed in SPSS v.18, *p* < 0.05 was considered to be significant.

## Results

### Qualitative assessment of dMRI-based indices

**Figure 2:**
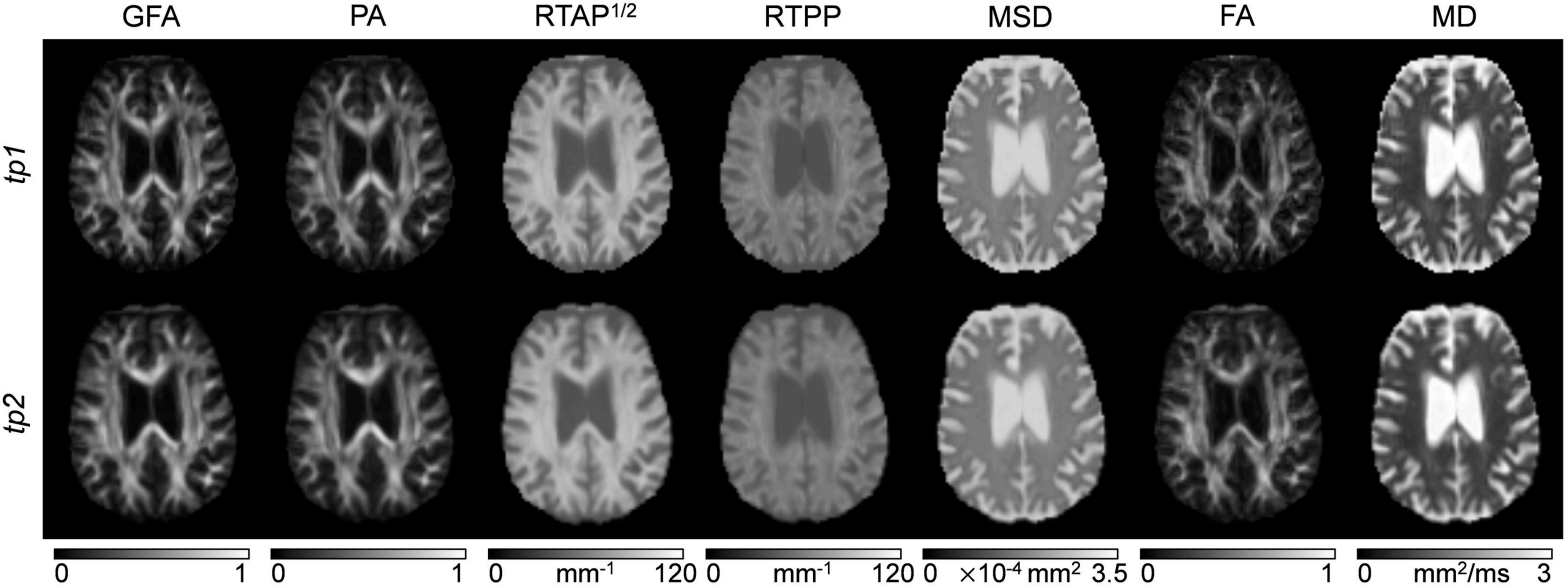
dMRI-based indices on a representative control. Axial slices of a representative control are reported for each index (columns) and each time point (rows). Images are displayed in radiological convention.

**Figure 3:**
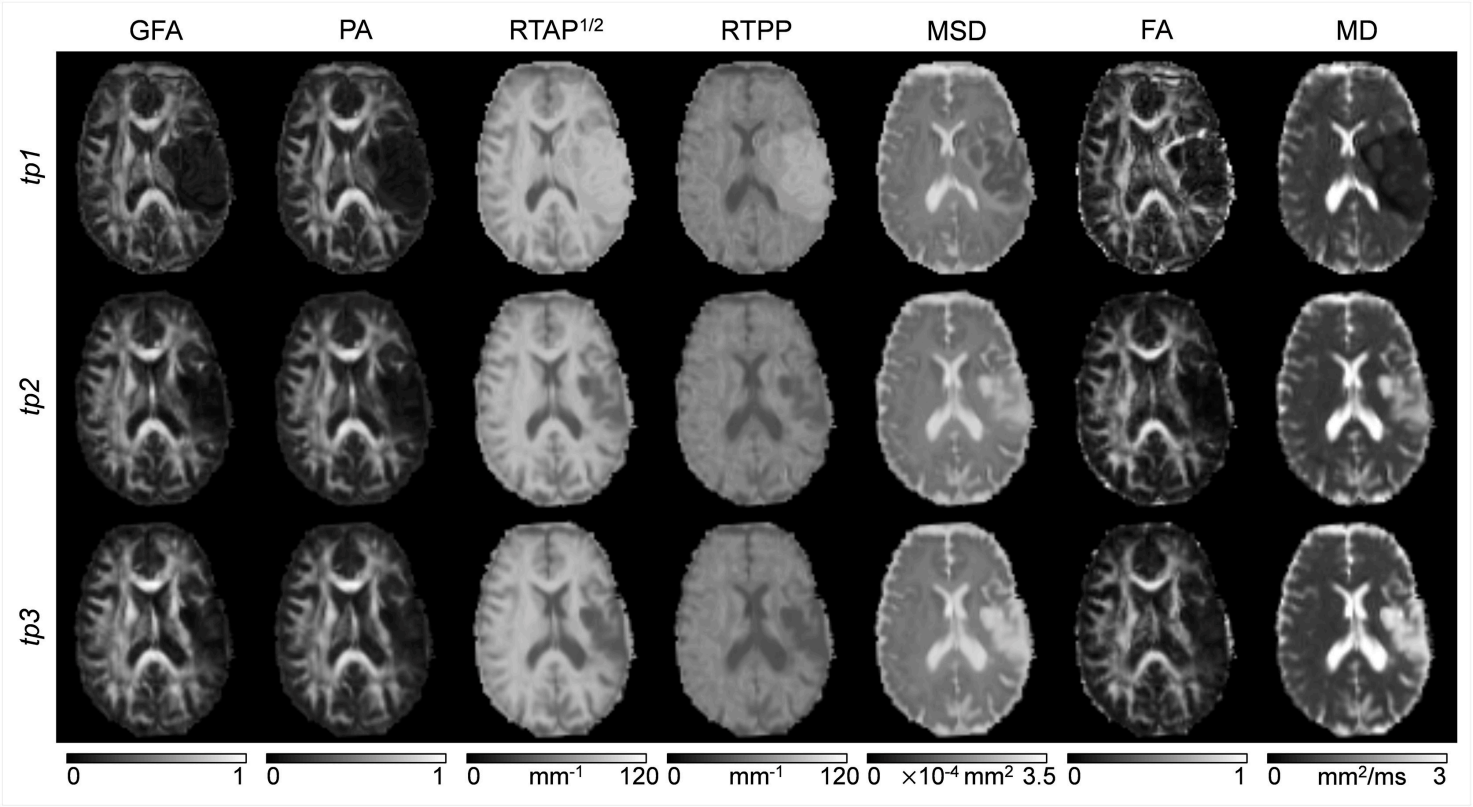
dMRI-based indices on a representative patient. Axial slices of a representative patient (ischemic stroke in left cortico-subcortical areas) are reported for each index (columns) and each time point (rows). Images are displayed in radiological convention.

Classical tensor-derived and 3D-SHORE-derived indices were estimated in all subjects and *tp*. Figures 2, 3 show the different maps calculated for each index across times in a representative control and a representative ischemic stroke patient, respectively. For ease of visualization and for the sake of clearer presentation, the three anisotropy measures were normalized to the respective maximum index value, while the square-root of the RTAP maps was extracted to report the values in the same range of RTPP, as in Avram et al. (2016).

All the anisotropy measures as well as RTAP and RTPP maps revealed high values in WM, while lower values were reached in GM and especially in voxels with strong CSF contribution. The opposite pattern was visible in MD and MSD maps, where WM appeared to be hypointense due to restricted diffusion while higher values were reached in GM and CSF tissues. These patterns were consistent across subjects and temporal scales. Comparing GFA, PA, and FA, both control and patient representative slices revealed a higher WM/GM contrast for the normalized 3D-SHORE-derived anisotropy measures that also appeared to be less noisy and more uniform throughout WM in comparison to the classical FA. Moreover, FA appeared to have lower values in regions with large fiber orientation dispersions where the single tensor representation precludes the possibility to cope with complex structures leading to drops. RTAP maps were hyperintense in regions of coherently packed WM fibers, while RTPP was similar in GM and WM tissues. Finally, MSD, and MD visually demonstrated a correlated behavior, appearing brighter in regions where water particles are free to diffuse like ventricles and darker in regions of restriction like WM.

In the stroke patient reported in Figure 3, a large ischemic lesion can be appreciated in the left hemisphere (cortico-subcortical areas) and the modulation of tissue microstructure is visible across the different *tp*. The lesion was hypointense in GFA, PA, MSD, FA, and MD at *tp1*, while markedly brighter than the other tissues in RTAP and RTPP. After 1 month from the injury (*tp2*), the contrast was reversed for these two indices, such that the lesion appeared hypointense as in the anisotropy measures, where hyperintensities within the lesion became visible in MSD and MD. Such a trend persisted at 6 months after the initial brain damage (*tp3*).

For all the subsequent quantitative analyses, we investigated the contralateral hemisphere only, where microstructural changes after stroke might be subtle and not visually detectable.

### Test-retest reproducibility on healthy controls

In terms of test-retest reproducibility, tract-based results highlighted excellent consistency across sessions in the three networks for tensor-derived as well as 3D-SHORE indices, with ICC > 0.8 in almost all cases and values close to unity for the SUBCORT loop (Supplementary Table 3). Indeed, the highest ICC was obtained for PA in SUBCORT (ICC = 0.96), followed by MSD in the same network (ICC = 0.95). Conversely, MSD together with RTPP reached the lowest values in CORT, although still amenable to be judged as having good reliability (ICC = 0.67 and ICC = 0.59, respectively). This high reliability was matched with high intra-subject stability across sessions as measured by CV_intra_ values, well below 10% and, in most of the cases, also below 5%. The lowest stability was found in the CC loop for MD (CV_intra_ = 7.7%), while MSD resulted to be the index with the highest stability in all the loops, reaching a remarkable 1.1% within-subject variability in the SUBCORT network.

GM region-based reproducibility results are reported in Table 2 in terms of mean and SD values across ROIs. RTAP, RTPP, MSD, and MD reached excellent consistency, with mean ICC > 0.90 and very low SD across ROIs (< 0.10). Conversely, all the anisotropy measures showed only good reliability and more variability across the different GM structures. This was further confirmed by the CV_intra_ measure, reporting mean values <10% in all cases albeit higher for GFA, PA, and FA in comparison to the other microstructural indices. Also in this case, MSD reached the lowest variability values with a limited spread around the mean.

**Table 2:** Reproducibility for gray matter (GM) outcomes.

	**ICC**	**CV_intra_ %**
GFA	0.63 ± 0.22	7.36 ± 2.96
PA	0.61 ± 0.24	6.82 ± 2.42
RTAP	0.91 ± 0.07	3.40 ± 1.63
RTPP	0.92 ± 0.07	1.73 ± 0.78
MSD	0.93 ± 0.09	1.97 ± 0.75
FA	0.66 ± 0.17	9.25 ± 3.59
MD	0.94 ± 0.08	3.09 ± 1.71

*Results are quantified in terms of intra-class correlation coefficient (ICC) and intra-subject coefficient of variation (CV_intra_) for all the indices. In particular, mean ± standard deviation values across all the considered GM regions are reported*.

**Figure 4:**
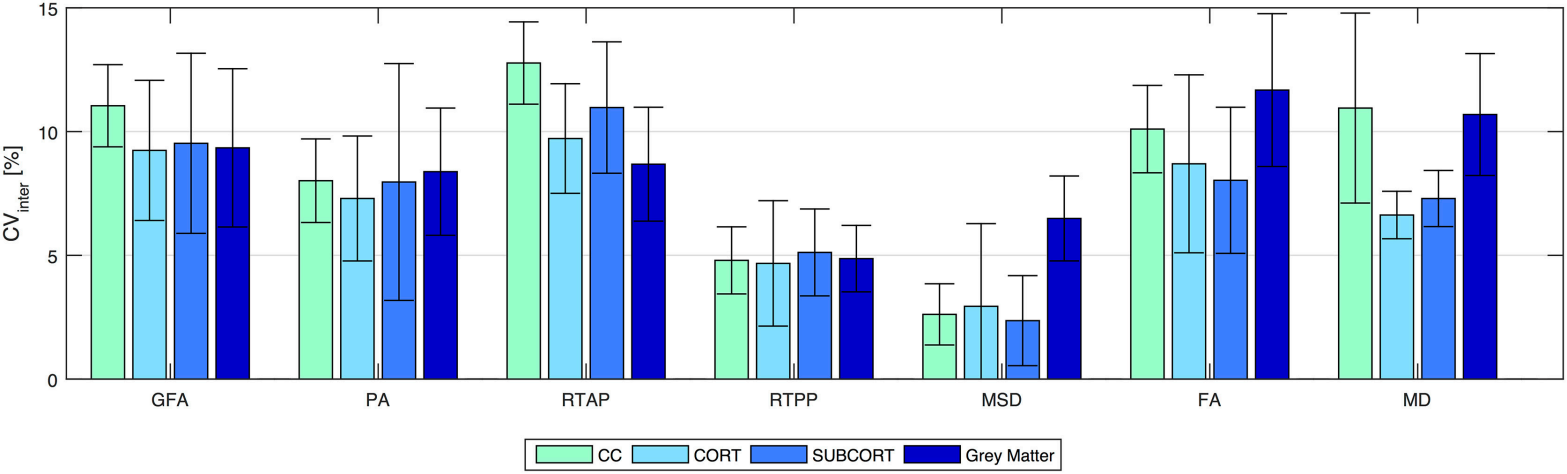
Reproducibility in terms of inter-subject coefficient of variation (CV_inter_) for all the indices and for all the outcome measures. Results are expressed as percentage and reported as mean ± standard deviation across connections (for tract-based) and regions (for region-based on gray matter), respectively. CC, transcallosal network; CORT, cortical network; SUBCORT, subcortical network.

Figure 4 shows the inter-subject variability results (CV_inter_) represented as mean ± SD across all the connections of a given loop for tract-based analysis, and across ROIs for region-based analysis on GM. As expected, the between-subject variability was higher than the within-subject, although the mean CV_inter_ values were ≤ 15% in all cases. Regarding the network analysis, similar patterns in the three loops were observed for each index, with RTPP and MSD featuring the lowest variability across subjects (RTPP: CV_inter_ = 4.67 ± 2.53 % in CORT; MSD: CV_inter_ = 2.36 ± 1.82 % in SUBCORT). Conversely, RTAP was the index showing more variability in all loops, especially in CC. The same trend was observed in the ROI-based analysis on GM, where the CV_inter_ values were similar to those resulting from tract-based analysis with RTPP and MSD reaching the highest stability (RTPP: CV_inter_ = 4.87 ± 1.34 %; MSD: CV_inter_ = 6.49 ± 1.72 %). It is worthy of note that all the values were within the recommended 15% range (Heiervang et al., 2006; Marenco et al., 2006), even though tensor-derived indices featured relatively lower stability across subjects in GM, with the highest values reached by FA (CV_inter_ = 11.68 ± 3.09 %).

### Quantitative assessment on tract-based outcomes–patients and controls

**Figure 5:**
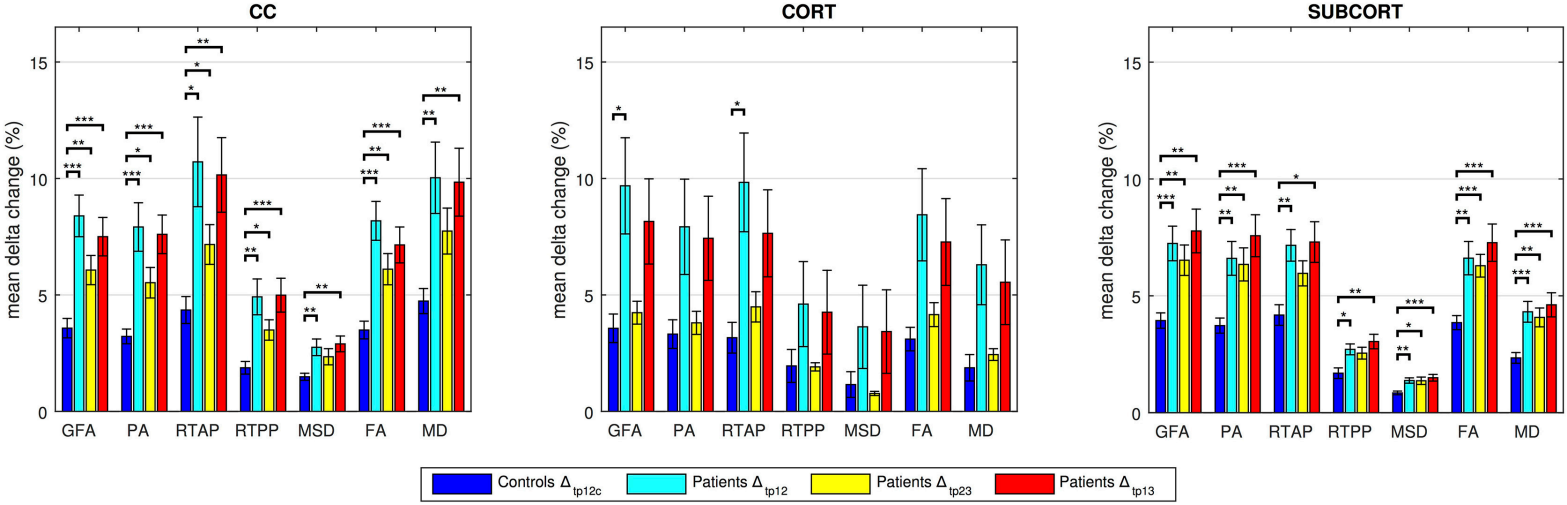
Group-based analyses on controls and patients over time. Longitudinal changes in percent absolute values in controls and patients are reported. The significant differences between cohort distributions are indicated in figure (*p < 0.05, ^**^p < 0.01, ^***^p < 0.001) for each index in transcallosal (CC), cortical (CORT), and subcortical (SUBCORT) networks. Mean ± standard deviation values across subjects are reported.

For each index and network, the mean of the percentage absolute changes between *tp* is reported in Figure 5 along with SD across subjects. The *p*-values resulting from the statistical analysis are shown as stars with three levels of significance (^*^p < 0.05, ^**^p < 0.01, ^***^p < 0.001). In all cases, data from the control group confirmed the limited percentage changes between time points, with mean values <5%, in agreement with the reproducibility results from the previous section.

Regarding the CC network, all the anisotropy measures (GFA, PA, and FA) reached the highest significance when comparing Δ_*tp*12*c*_ and Δ_*tp*12_ as well as Δ_*tp*12*c*_ and Δ_*tp*13_ (*p* < 0.001). Moreover, GFA and FA showed higher significance than the other microstructural indices in the comparison between Δ_*tp*12*c*_ and Δ_*tp*23_ (*p* < 0.01). MSD and MD highlighted the same patterns across time and the same statistical differences, with no significant changes between Δ_*tp*12*c*_ and Δ_*tp*23_. In the CORT network, only few significant differences were detected between controls and patients (Δ_*tp*12_) by GFA and RTAP, while for all the other indices the longitudinal changes, although appreciable, did not reach the statistical threshold. Conversely, several significant differences were detected again in the SUBCORT loop by all the indices at multiple time scales, except for RTAP and RTPP which did not depict significant changes between Δ_*tp*12*c*_ and Δ_*tp*23_. All the anisotropy measures confirmed the presence of marked changes over time involving also this network, with similar patterns to the findings shown in CC.

**Figure 6:**
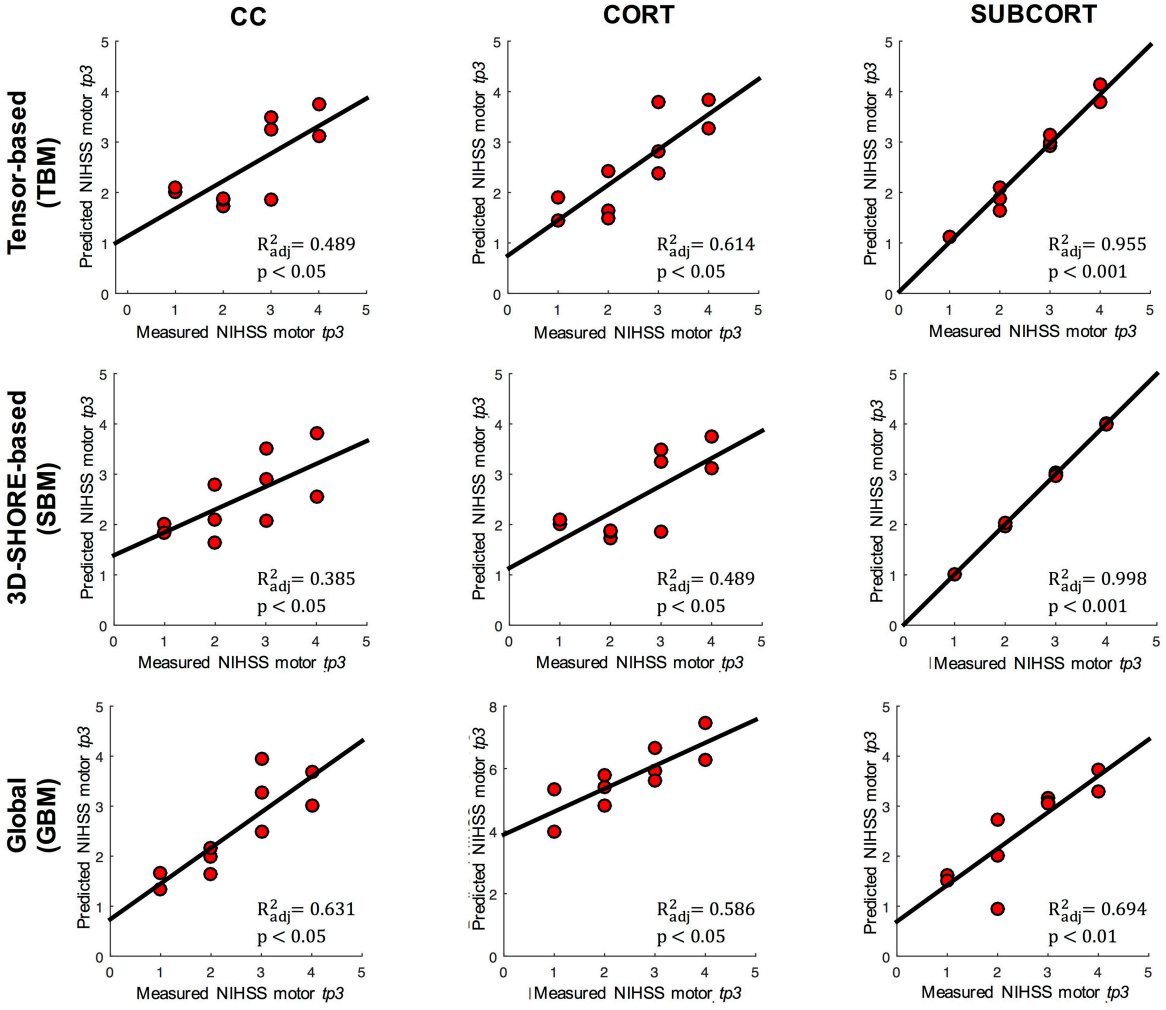
Linear regression models. Representation of the measured and predicted NIHSS-motor score at *tp3* using tensor-based (TBM), 3D-SHORE-based (SBM), and global (GBM) predictive models. For each model, the adjusted *R*^2^ and corresponding *p*-values are reported.

Extending the preliminary analyses on predictive models reported in Brusini et al. (2016), the tract-based results in patients were further used to predict the clinical motor outcome at *tp3* by relying on several regression models. The reference linear regression model including only clinical variables at baseline (age, stroke size and NIHSS motor score at *tp1*) and avoiding microstructural indices could predict the NIHSS outcome at *tp3* with low correlation (*R*^2^ = 0.546; adjusted *R*^2^ = 0.489; *p* < 0.05). The TBM, enclosing MD-FA at *tp1* plus the clinical variables, allowed increasing the prediction capability of the reference model in the CORT and SUBCORT networks (Figure 6, first row). In detail, the TBM for SUBCORT presented the best performance (*R*^2^ = 0.975; adjusted *R*^2^ = 0.955; *p* < 0.001) holding MD, FA, stroke size and age as relevant predictors. In the case of the CORT network, a higher correlation than the reference model was found with the TBM retaining only stroke size and MD as significant predictors (*R*^2^ = 0.700; adjusted *R*^2^ = 0.614; *p* < 0.05). Conversely, the TBM for CC did not include any microstructural index, returning the reference model as the optimal one.

The SBM, embedding the five 3D-SHORE indices at *tp1* plus the clinical variables, reached the highest correlation in the SUBCORT network (*R*^2^ = 1; adjusted *R*^2^ = 0.998; *p* < 0.001) (Figure 6, second row). The optimal predictive model held clinical variables plus GFA, MSD, RTPP, and PA as significant predictors. The SBM for CORT excluded all the microstructural indices, leading to the reference model as the optimal one. Finally, in the CC network the SBM presented a slightly lower correlation than the reference (*R*^2^ = 0.454; adjusted *R*^2^ = 0.385; *p* < 0.05) but highlighting RTPP as the only significant predictor.

The GBM, including only the dMRI-based indices, allowed to substantially increase the capability to timely predict the motor outcome compared to the clinical reference model (Figure 6, third row). In detail, the SUBCORT network provided again the highest correlation (*R*^2^ = 0.728; adjusted *R*^2^ = 0.694; *p* < 0.01) keeping only RTPP as significant predictor. The predictive model for the CC network also featured high correlation (*R*^2^ = 0.713; adjusted *R*^2^ = 0.631; *p* < 0.05) maintaining MD and RTPP as predictors, while GFA, RTAP, and MD were retained in the predictive model for CORT. This network led to the GBM with the lowest correlation (*R*^2^ = 0.724; adjusted *R*^2^ = 0.586; *p* < 0.05), but still higher than the reference model. Further details on the predictive models and the retained predictors are reported in the Supplementary Tables 4.

### Quantitative assessment on GM region-based outcomes–patients and controls

**Figure 7:**
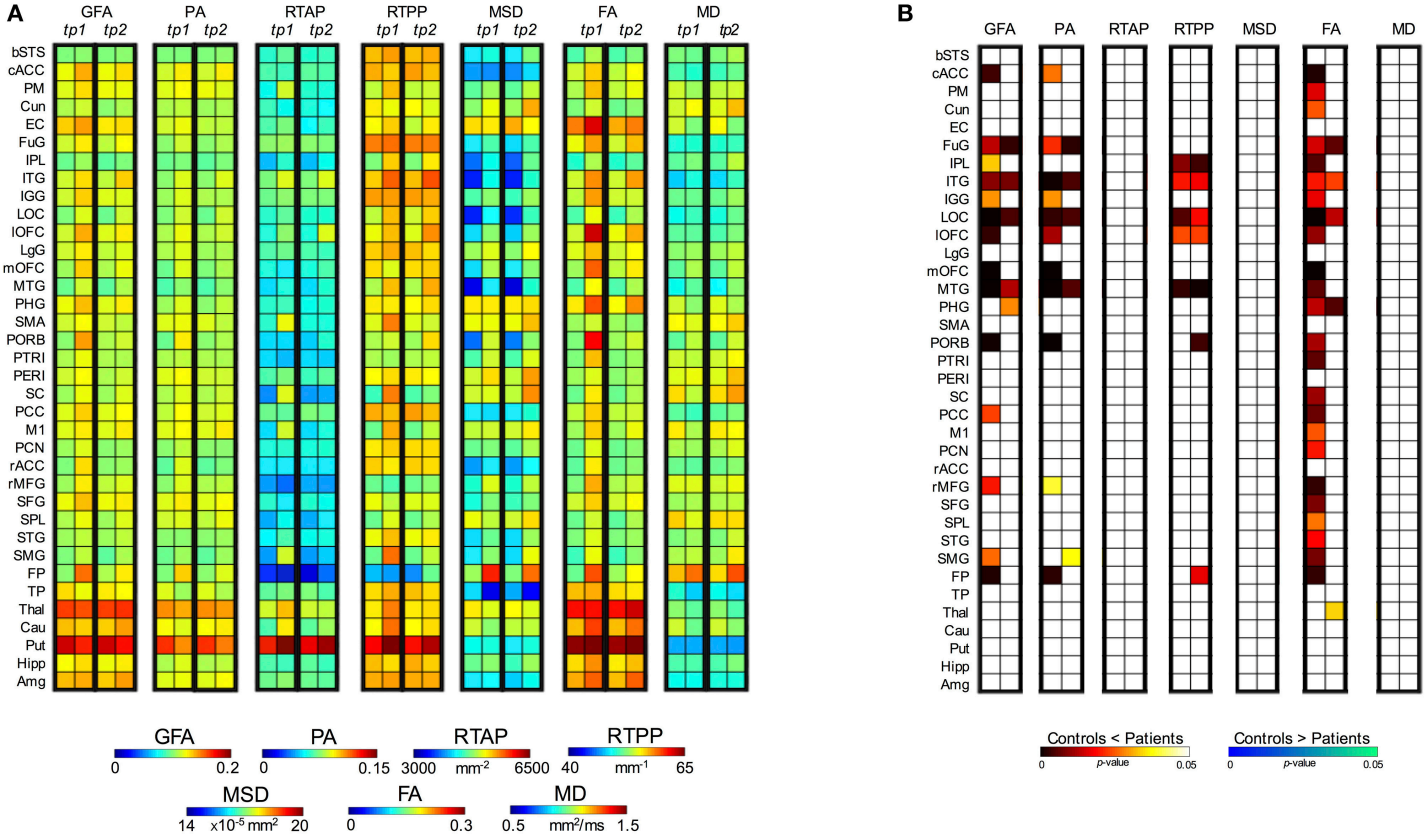
*Post-hoc* test results for the three-way mixed ANOVA (controls vs. patients). **(A)** For each index and each time point (*tp*) block, the first column represents the mean values for the controls while the second column the mean values for the patients. **(B)** *Post-hoc* results expressed in terms of *p*-values for the significant interactions between Group, TP and Region (ROI). Two different colormaps are used to display the *p*-values for the ROIs with significant differences between control and patient mean values (hot: Controls < Patients; cold: Controls > Patients). These values (*p* < 0.05) are Bonferroni corrected for multiple comparisons.

Regarding the control vs. patient analyses on the outcomes from the region-based quantification in GM tissues, the mixed ANOVA revealed a significant three-way interaction between Group, Time (TP) and Region (ROI) for all the anisotropy measures (GFA, PA, and FA) and RTPP. Details about these statistical results are reported in Table 3. For the four indices, *post-hoc* Bonferroni tests revealed significant between-group differences in several regions at both time scales, showing in these cases higher values in patients than controls (Figure 7). While the most widespread changes were detected in terms of anisotropy at *tp1*, four common regions were identified as significantly altered (Patients > Controls) also by RTPP. In detail, the inferior temporal gyrus (ITG) and the lateral occipital cortex (LOC) were in common at both *tp*, while the lateral orbitofrontal cortex (lOFC) and the middle temporal gyrus (MTG) presented high significance (*p* ≤ 0.01) at *tp1* and *tp2* in GFA, PA, and RTPP and only at *tp1* in FA (Figure 7). RTPP changes were more visible at *tp2*, with several regions showing higher values in patients compared to controls and non-significant anisotropic differences. The remaining indices failed to reach a significant three-way interaction even though control vs. patient differences can be visually appreciated in Figure 7A. In particular, for RTAP a similar trend to the anisotropy measures was detected in all the regions, especially at *tp1* over motor areas and subcortical nuclei as PM, SMA, SC, M1 and Thal, Cau and Put (Patients > Controls). For MSD, while few ROIs presented relatively higher values in patients at *tp1*, there was an overall increase in all regions at *tp2* (Patients > Controls), except for the temporal pole where lower values were found over time in this group. Finally, MD patterns were in line with MSD results, although with less marked changes between groups.

**Table 3:** ANOVA results (three-way mixed ANOVA) for the control vs. patient comparison of gray matter outcomes.

	**Between-subject**		**Within-subject**			
	**Group**		Group^*^ROI		Group^*^TP^*^ROI	
	**F-ratio (1, 18)**	***p*-value**	**F-ratio (35, 630)**	***p*-value**	**F-ratio (35, 630)**	***p*-value**
GFA	6.205	0.023^*^	2.340	<0.001^*^	2.235	<0.001^*^
PA	6.256	0.022^*^	2.218	<0.001^*^	1.669	0.010^*^
RTAP	1.548	0.229	1.249	0.157	1.326	0.102
RTPP	2.064	0.168	2.152	<0.001^*^	1.843	0.003^*^
MSD	2.681	0.119	2.601	<0.001^*^	0.552	0.990
FA	7.346	0.014^*^	2.082	<0.001^*^	2.731	<0.001^*^
MD	0.186	0.671	1.825	0.003^*^	1.105	0.314

The three independent variables were Group (between-subject factor), Time Point (TP) and Region (ROI) (within-subject factors), while the dependent variable was the mean index value. Group, along with Group*ROI and Group*TP*ROI interactions, are expressed in terms of F-ratio (degree of freedom, error) and p-values.

* *, significant values*.

Moving a step backward in the mixed ANOVA, all the indices except RTAP revealed a significant two-way interaction between Group and ROI confirming that, considering the overall time scales, there were differences in specific GM regions between the two groups (Table 3; Supplementary Figure 1). The anisotropy measures were highly consistent, with FA highlighting more widespread increased values in GM for patients as before. Finally, only GFA, PA, and FA revealed an overall significant main effect of Group (between-subject factor), as reported in Table 3.

**Figure 8:**
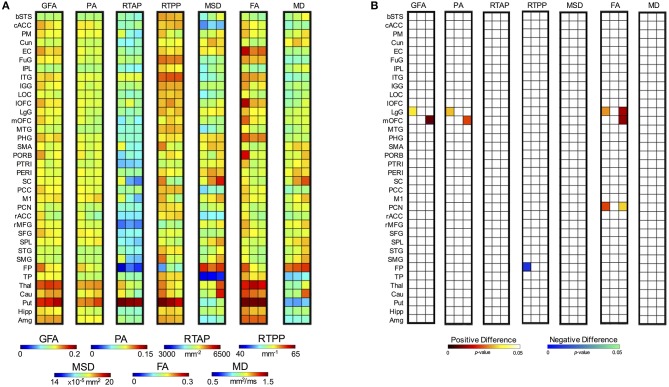
*Post-hoc* test results for the two-way ANOVA for repeated measures on patients. **(A)** For each index, the first column represents the mean values at *tp1*, the second column at *tp2* and the third at *tp3*. **(B)** *Post-hoc* results expressed in terms of *p*-values for the significant interactions between Time Point (TP) and Region (ROI). Each column in the matrix refers to a specific statistical comparison between time scales, i.e. *tpi* vs. *tpj* with *i* = *1, 2* and *j* = *2, 3* (first: *tp1* vs. *tp2*; second: *tp2* vs. *tp3*; third: *tp1* vs. *tp3*). Two different colormaps are used to display the *p*-values for the ROIs with significantly different values between the considered time scales (hot: positive difference, *tpi* > *tpj*; cold: negative difference, *tpi* < *tpj*). These values (*p* < 0.05) are Bonferroni corrected for multiple comparisons.

Considering the longitudinal analysis on the patient measures only, again all the anisotropy indices along with RTPP and MD revealed a significant interaction between TP and ROI. In details, for GFA *F*_(70, 630)_ = 1.61, *p* = 0.002; for PA *F*_(70, 630)_ = 1.52, *p* = 0.006; for RTPP *F*_(70, 630)_ = 1.47, *p* = 0.01; for FA *F*_(70, 630)_ = 1.92, *p* < 0.0001; and for MD *F*_(70, 630)_ = 1.76, *p* = 0.0003 (Supplementary Table 5). *Post-hoc* Bonferroni tests (Figure 8) highlighted for the three anisotropy measures consistently significant differences over the lingual gyrus (LgG) for *tp1* vs. *tp2*, and in the medial orbitofrontal cortex (mOFC) for *tp1* vs. *tp3*. Moreover, FA presented LgG differences for *tp1* vs. *tp3*, and in the precuneus (PCN) for both *tp1* vs. *tp2* and *tp1* vs. *tp3*. In all these statistically significant changes, higher values were detected just after the stroke event (*tp1*) in comparison to *tp2* and *tp3*. Conversely, an opposite trend was found for RTPP detecting a single region [frontal pole (FP)] with higher values at *tp2* compared to *tp1*. For MD, despite the significant interaction no regions survived the Bonferroni corrections of the *post-hoc* paired tests (Figure 8). When using a less conservative approach [Least Significant Different (LSD) *post-hoc* tests], five regions, including PM, SC, and Thal, turned out to be significantly increased at *tp3* compared to *tp2* and *tp1* (Supplementary Figure 2). Applying LSD *post-hoc* tests also to the other indices, the anisotropy measures revealed more widespread regions of increased values in the early phase (*tp1*) in comparison to the other two time points, consistently with the results from the mixed ANOVA. GFA and PA, in addition, showed higher values at *tp3* compared to *tp2* over two motor regions, e.g., Put and M1, respectively. Finally, RTPP confirmed a significant increase over time (both *tp2* and *tp3*) in comparison to *tp1* in the FP region.

Regarding the other two indices that did not show a significant interaction (RTAP and MSD) and were thus precluded to be evaluated with *post-hoc* tests, a different trend was visible across time with a series of appreciable longitudinal differences (Figure 8A). In particular, RTAP revealed a similar behavior to the anisotropy measures, with higher values at *tp1* that decreased over time, especially at *tp3*. Conversely, MSD highlighted higher values over time, as in the case of MD, with marked visual increases at *tp3* over several regions (as PM, SC, FP, Thal, Put, Cau).

## Discussion

In this study, our results suggest that 3D-SHORE-based microstructural descriptors obtained from DSI data are capable to quantify the remodeling of WM tracts and GM regions involved in motor recovery after ischemic stroke. 3D-SHORE-based indices proved to perform similarly to the classical DTI indices (FA and MD) and revealed common patterns across the networks and ROI evaluated in the analyses.

Considering their performance and different nature, their combination in clinical studies would allow to provide a more detailed and specific tissue characterization, allowing to disentangle different conditions where tensor-based indices take the same values. For instance, DTI cannot distinguish between a reduction of FA caused by crossing fibers and one caused by a decrease of neural density in a voxel. Conversely, the joint exploitation of RTAP and RTPP can allow disentangling such ambiguity, as RTAP and RTPP both diminish in the case of neuronal density reduction, while RTAP decreases and RTPP increases for crossing fibers, as previously reported (Zucchelli et al., 2016a). In addition, the combination of tensor-based and SHORE-based indices in the linear regression models allowed to greatly increase their ability to predict the clinical motor outcome in all the considered networks. To the best of our knowledge, this is the first study focusing on the quantitative comparison between 3D-SHORE-based and tensor-based descriptors in healthy subjects and in a patient population, aiming at demonstrating their behavior in different brain conditions/tissues and accomplishing an essential step toward their applicability as viable tissue markers.

### Qualitative assessment of dMRI-based indices

A growing body of literature is currently reporting the advantages of using multiple b-values in terms of both detecting fiber crossings (Sotiropoulos et al., 2013; Jeurissen et al., 2014) and recovering the tissue microstructure (Assaf and Basser, 2005; Zhang et al., 2012; Kaden et al., 2016). Because of these facts, nowadays, sampling schemes presenting higher b-values (as DSI and multi-shell) are becoming popular in research and started to appear also in clinical application. In order to fully exploit advanced dMRI datasets, reconstruction models that require multiple b-values such as the 3D-SHORE are necessary and therefore will become more common in this field. In this context, it is therefore necessary to provide an extensive characterization of these indices in describing tissues in physiological and pathological condition, as we did for stroke patients. In line with the findings firstly described by Özarslan et al. (2008, 2013), our results suggest that 3D-SHORE-based indices can provide a wide set of information, reflecting meaningful tissue properties as visually appreciable from the different maps. In particular, the values estimated in our healthy population for each index and their spatial distribution across the different anatomical structures appear to be in agreement with the available literature results (Özarslan et al., 2013; Avram et al., 2016; Zucchelli et al., 2016a), with a high consistency across subjects and time. These 3D-SHORE-based metrics are able to provide accurate microstructural information especially in brain regions characterized by complex architectures and geometries, to which the classical indices have low sensitivity. GFA and PA represent alternative measures of anisotropy to the classical FA, based on different mathematical formulations. Indeed, while GFA is a measure of the ODF variance, PA is derived from the EAP as a measure of its deviation from the isotropic component, and FA is computed from the tensor eigenvalues. In consequence, they provide different descriptors of the diffusion anisotropy with a high degree of correlation. However, GFA and PA are able to more properly quantify the anisotropy, presenting more contrast between the GM and regions with multiple fiber crossings in which the FA usually results in the same value. The two zero-displacement probability measures derived from SHORE reflect diffusion restriction in different directions, respectively radially (RTAP) and axially (RTPP) to the main diffusion direction (Özarslan et al., 2013). Consistently, RTAP maps exhibited high values in regions of coherently packed WM fibers, as the corpus callosum which is less contaminated by partial volume effects. RTPP values were similar in both GM and WM tissues featuring less WM/GM contrast. This could suggest similar apparent axial diffusivity for WM and GM, even though the mapping of this measurement to real tissue microstructural properties is still an open issue. Finally, MSD and MD were consistently higher in regions featuring free diffusion, like the CSF and in areas with ischemic oedema (Alexander et al., 2007). These two indices are directly related via the Einstein diffusion equation as reported in the works of Wu and Alexander (2007) and Hosseinbor et al. (2013) and, accordingly, are visually correlated.

Evaluating qualitatively the longitudinal maps derived from the stroke patients, the microstructural indices exhibited a different behavior in the voxels belonging to the damaged area but with a consistent pattern. Indeed, while all the anisotropy measures revealed low values within the lesion that persisted over time, RTAP and RTPP shifted from initial hyperintensities toward hypointensities after 1 month from the event (*tp2*), highlighting an opposite trend for anisotropy and restriction. This stresses the complementarity of the information brought by those indices. Furthermore, considering their opposite trend in comparison to MSD and MD (from hypo- to hyperintensities) and the ischemic nature of the stroke, these findings support the hypothesis of Avram et al. (2016) according to which the zero-displacement measures are more specific biomarkers of the presence of restricting barriers to diffusion. Interestingly, RTAP and RTPP featured the highest values at *tp1* highlighting restricted diffusion in the lesion. Moreover, we found MSD to be more contrasted than MD inside the ischemic lesion in all cases. In particular, this index seems to identify and characterize different portions of the lesion, while MD appears to be more homogeneous in the same areas. Some patients (mainly those with extensive lesions) also revealed increased MSD values in the periphery. However, as this pattern was not confirmed in all cases, a larger sample size and more focused analyses on the stroke lesion would be necessary to draw robust conclusions on this aspect, possibly pointing at an inflammatory reaction which has been previously described (Wang et al., 2007; Kim et al., 2016). Finally, the heterogeneous patterns of RTAP, RTPP, and MSD visible within the lesion 1 week after stroke could be of help for distinguishing the ischemic core from the penumbra area. This issue deserves further investigation.

### Reproducibility analyses on controls

The quantitative analysis of possible plasticity processes was focused on the contralateral hemisphere to the stroke. The contralesional GM and WM tissues have been widely considered as normal appearing, although the plasticity and compensatory processes that might take place in the non-injured areas are still not well understood. First of all, several complementary aspects were evaluated on healthy controls in order to quantify the reliability of these microstructural indices through a test-retest paradigm and their potentialities as novel biomarkers for stroke recovery. In particular, both 3D-SHORE-based and DTI indices exhibited high reproducibility, as quantified by ICC, and high stability, as quantified by intra/inter-subject CV parameters, on both tract and region-based outcomes.

Interestingly, for tract measures the 3D-SHORE index MSD, rarely considered in previous studies, showed the lowest intra-subject variability (CV_intra_) in all cases, and the highest reliability (ICC) in CC and SUBCORT. Conversely, it revealed lower, although still good, ICC values in CORT along with RTPP that resulted to be the index with the lowest reliability in this network. This is possibly related to the fact that these two indices exhibited here a relatively higher within-subject SD for repeated measurements than in the other cases, which resulted to be closer to the between-subject SD values and therefore led to lower ICC values for this loop. Despite this consideration concerning the CORT loop only, the reliability and discriminative power of MSD and RTPP were not compromised as further proven by the other group-based analyses performed in this study. To note that beside Brusini et al. (2016), where some of these indices were initially evaluated along WM tracts, no other studies have quantified the reproducibility of 3D-SHORE-based metrics across subjects and sessions. Moreover, the previous reports aiming at quantifying the reliability of classical tensor-based measures generally focused only on few major fiber tracts (e.g., corpus callosum, cingulum, fornix and arcuate fasciculus) (Heiervang et al., 2006; Danielian et al., 2010; Wang et al., 2012) rather than considering specific brain networks with different sets of tracts. Despite this main difference, our findings are in line with the results of these studies, which demonstrated reliable measurements for FA and MD featuring both inter-session CV_intra_ ≤ 10% and ICC ≥ 0.70, with some variability related to the considered tract.

Regarding region-based outcomes, the reproducibility analysis in GM ROIs revealed a higher intra-subject variability for the three anisotropy measures (GFA, PA, and FA) in comparison to the other indices, with mean values still well within the 10% range, matched with a good reliability from ICC. This is possibly due to the lack of directed orientation in a tissue as GM (Basser and Ozarslan, 2009) and is in agreement with previous studies showing a two-three times higher variation of FA in regions of GM compared to WM structures (Vollmar et al., 2010; Bouix et al., 2013). Conversely, MSD and RTPP appeared again as featuring the lowest intra-subject variability and, along with MD, reached the highest ICC reliability values. The performance of FA for GM ROIs appears to be in line with previous reports evaluating DTI indices in this tissue (Veenith et al., 2013; Grech-Sollars et al., 2015), showing higher CV_intra_ values for the whole GM than for MD (8–11% vs. 2–5%, respectively) and a wide range of variation across the different GM structures (3.3–19.2%). Conversely, no studies have previously quantified the measurement precision of 3D-SHORE-based indices in GM regions, therefore our findings add an important step to the current literature on the topic and their reassurance in terms of reliability encourages their use for evaluating GM tissues as well.

Considering as additional reliability measure the between-subject variability, we found average CV_inter_ values well below the 15% threshold for both tract- and region-based outcome. Among the seven variables, RTPP and MSD generally had lower CV_inter_ than the other metrics with average values ≤ 6%. Tensor-based measures revealed overall lower between-subject stability than 3D-SHORE-based indices, especially in the GM ROIs where the average values were around 10%. Previous studies have indicated FA and MD as the measures with lower CV_inter_ in different WM fiber tracts, for example Wang et al. (2012) reported average values in the range 2.4–7.6% for FA and 1.7–9.9% for MD respectively, while Grech-Sollars et al. (2015) showed mean inter-subject values <6% for the whole GM and WM regions (not tracts). Our results confirmed the good inter-subject stability for FA and MD but demonstrated that the 3D-SHORE-based indices improve on the classical measures in terms of between-subject variability in most of the cases. The latter observation demonstrated the gain in using a multi-b-values model such as 3D-SHORE. In particular, GFA and MSD were already defined the analogs of FA and MD for multi-b-values acquisitions by Hosseinbor et al. (2013). The combined high stability over time, relatively higher inter-subject variability (CV_intra_ < < CV_inter_) shown by the 3D-SHORE based indices, which is a pattern that can help detecting group differences between subjects, and excellent inter-session ICC values for most of the cases reinforce their potentialities as microstructural biomarkers for revealing longitudinal changes.

### Quantitative analyses on tract-based outcomes of WM

Longitudinal group-based analyses were performed to statistically compare the mean absolute changes between time points calculated for each network. Regarding 3D-SHORE-based indices, the Bonferroni corrected *t*-tests revealed several highly significant differences between patients and controls in the SUBCORT and CC networks, also for the newly introduced MSD index. These findings further confirm and strengthen our preliminary results on a subset of 3D-SHORE indices (Brusini et al., 2016), where the *t*-tests were corrected for multiple comparisons with FDR. Conversely, a more conservative correction was employed here in order to quantify with additional confidence the longitudinal changes detected by the different indices and to reduce false positive results. Tensor-derived indices also exhibited similar patterns to 3D-SHORE descriptors, in terms of both evolutions of changes over time and level of significance.

In all cases, the highest levels of significance were reached in the patient group for the *tp1-tp2* and *tp1-tp3* relative changes, suggesting the presence of marked modifications in the contralateral hemisphere just 1 week after the stroke event (*tp1*). Interestingly, 3D-SHORE-based indices appeared to be the only capable of depicting statistically significant changes across the CORT loop. Indeed, only GFA and RTAP found a significant patient vs. control difference in the first phase (*tp1-tp2*), further highlighting the relevance of this time scale in the course of the disease.

These findings are in line with the few previous works reporting changes in the WM tracts of the contralesional hemisphere after stroke. Indeed, the possible modifications in the contralateral hemisphere with respect to the lesion have been scarcely investigated in literature, especially in humans, as these tissues have been widely disregarded as considered healthy and not directly involved in any rearrangement process (Maniega et al., 2004; Ozsunar et al., 2004). However, as the field moved forward, it became apparent that also the non-injured hemisphere undergoes marked changes and has a fundamental role in stroke recovery, as recognized by several authors relying on different MRI techniques (Ward et al., 2003; Gerloff et al., 2006; Crofts et al., 2011; Granziera et al., 2012b; Lin et al., 2015). Specifically, Crofts et al. (2011) showed how communicability values, derived from complex network analysis, were reduced in both ipsilateral and homologous contralateral regions. Moreover, Granziera et al. (2012a) reported significantly increased apparent diffusion coefficient (ADC) values in the infarct region (in both GM and WM tissues) moving from acute to chronic, whereas WM FA significantly decreased in the mirror regions. Our study extends the available literature on the topic and the novel biomarkers derived by the 3D-SHORE model possibly add new metrics that can be employed in this context (for a detailed overview see Kim and Winstein, 2017).

In addition, the predictive power of all the microstructural indices for patient motor outcome at *tp3* were investigated relying on the tract-based values and comparing several regression models for the prediction. Notably, among the three loops, the SUBCORT was the only one for which all the three types of models created (tensor-based model, 3D-SHORE-based model, global microstructural model) reached excellent performance. In particular, the 3D-SHORE-based model, combining a subset of these indices together with clinical patient information, led to the best linear regression model featuring a very high predictive power ($R_{\text{adj}}^{2}$ = 0.998, *p* < 0.001), which slightly outperformed the optimal model we found in our previous work ($R_{\text{adj}}^{2}$ = 0.988, *p* = 0.009) (Brusini et al., 2016). The set of indices in the optimal model of this work embeds MSD, suggesting that this index holds a higher potential in probing stroke-induced microstructural changes during the early phase.

The model using all the microstructural indices led to the best performance in the SUBCORT loop, reaching the highest correlation score ($R_{\text{adj}}^{2}$ = 0.694, *p* < 0.01) and keeping RTPP as key predictor. The relevance of RTPP for subcortical WM tracts appears to be coherent with another observation of Avram et al. (2016) according to which RTPP is very sensitive to deep structures, showing higher intensity in nuclei like thalamus. RTPP also highlighted high predictive power in CC, contributing to the optimal model for both the 3D-SHORE-based and global model, in combination with MD in this latter case. These results, jointly with the high precision and the ability to detect significant changes between patients and controls, stress the potential of this index in the considered task.

### Quantitative analyses on ROI-based outcomes of GM

Besides evaluating the performance of the different indices along the WM connections of specific brain networks, we performed a quantitative comparison of their patterns within contralateral GM regions. GM tissue changes related to the disease are generally quantified by volume or density analyses and are very rarely investigated with dMRI-based indices. A growing body of literature is emerging to endorse the use of dMRI techniques for detecting microscopic changes in GM in different disorders. Indeed, the analysis of diffusivity GM changes using MD has shown to be promising for detecting abnormalities in Alzheimer disease (Weston et al., 2015) and multiple sclerosis (Ceccarelli et al., 2007). GM FA alterations were also demonstrated in schizophrenic patients in Situ et al. (2015), reporting increased MD and decreased FA values in patients compared to controls. In stroke patients, studies in GM are less consistent and generally consider the tissues in the contralateral hemisphere as normal appearing, although regions remote (upstream or downstream) from the infarct have been demonstrated to undergo marked changes over a time course of 2 days to 1 year (Sotak, 2002). In one of these studies using the contralateral part as reference, Maniega et al. (2004) showed a trend of increased MD/decreased FA values within the lesion, which just started the first week from the event.

In our study, the longitudinal analyses on the patient group demonstrated a similar pattern but in the contralateral hemisphere, revealing an increase in MD values over time which mainly involved GM motor regions. Conversely, FA exhibited an initial widespread increase at *tp1* over temporo-frontal and motor areas, followed by a gradual decrease toward normality at *tp3*. This was further confirmed by the group-based comparisons with ANOVA, highlighting in most of these regions significantly higher FA values at *tp1* in patients vs. controls, whereas the increased pattern remained restricted to few ROIs when *tp2* values were evaluated. Similar patterns of alterations were detected also by SHORE-based indices, in particular by GFA, PA, RTPP and MSD. The group comparisons 1 week after the stroke revealed several GM regions (cACC, FuG, IGG, mOFC, PORB, rMFG, FP, ITG, LOC, IOFC, MTG) in which the patients exhibit significantly higher values for all the anisotropy indices (GFA, PA, FA) with respect to the controls. Considering that in the same regions, at the same time point, the MD and MSD appear to be increasing (Figure 7A), although not significantly, we can speculate that we are observing a general increase of the diffusivity along the main diffusion direction in the GM. More difficult to interpret is the simultaneous increase of the RTPP in some of these regions (ITG, LOC, IOFC, MTG). RTPP is generally inversely proportional to anisotropy in WM, e.g., RTPP is low in single fiber bundle areas such as the CC, and higher in crossing regions (Özarslan et al., 2013; Avram et al., 2016; Zucchelli et al., 2016a). Understanding the possible causes of this contemporary increase of RTPP and anisotropy in the GM will be one of the aims of our future works.

Contralateral changes in GM involved not only regions in the motor systems, but also areas playing an important role in cognition and behavior, as the FP and frontal areas, supporting the hypothesis of extensive rearrangements during stroke recovery. These indices therefore confirm their potentialities in describing not only WM but also GM properties, with high reliability and discriminative power. However, RTAP and MSD, which resulted to be suitable to characterize WM tracts in all the networks, appeared to be less sensitive to GM changes. Indeed, these indices failed to highlight statistically significant differences in the GM areas, especially when comparing the patient data over time. However, they deserve further investigations considering their good stability over time and their physiological relevance.

It is worth mentioning that the impact of partial volume effects was minimized by restricting the analysis to voxels where the GM contribution was above the 95%. This further enhances the hypothesis of extensive contralateral changes involving also the GM, reducing the contamination by other tissues.

As a side note, we also extracted for each patient and time point the average volumes of GM ROIs (results not shown). However, when statistically compared by means of a two-way repeated measure ANOVA, no significant changes were detected, possibly because of the small sample size and the limitations of such morphometric measure that might be not sensitive enough to subtle changes in the contralateral hemisphere. A larger sample size and more sophisticated analyses, for example based on cortical thickness measures or voxel-based morphometry, might be more suitable for depicting GM longitudinal changes following stroke, as often done in literature (Stebbins et al., 2008; Brodtmann et al., 2012). Our results, though preliminary, support the hypothesis that SHORE-based indices might hold the potential of revealing GM plasticity processes in the contralesional stroke area. We are aware of the fact that the interpretation in terms of geometrical restriction of the diffusion of the SHORE-derived indices in GM is prone to criticism because the real tissue architecture cannot be directly mapped to the underlying reference model (i.e., the pore). However, the fact that differences across time within a patient population and across groups can be detected using such indices provide evidence in favor of their exploitability as potential numerical biomarkers for GM plasticity in disease, leaving their interpretation in terms of microstructural properties an open issue.

Some limitations have to be acknowledged. This work has to be considered as a preliminary comparison between DTI and SHORE-based EAP derived indices in stroke. Here, we considered only the two most used DTI derived indices (FA and MD) and some of the principal EAP derived indices (RTAP, RTPP, MSD, PA, GFA). However, it will be interesting to extend the analyses to further indices that can be derived, e.g., the radial and axial diffusivity for the DTI, RTOP and the MAP-MRI non-gaussianity for the EAP. Moreover, our findings are based on the comparison between 10 healthy subjects and 10 ischemic stroke patients. A higher number of subjects would be necessary in future studies to fully exploit the potentialities and discriminative/predictive power of these rather novel indices. In particular, the linear regression analyses have to be carefully evaluated bearing in mind they are preliminary, although encouraging, findings. Indeed, the limited sample size precluded the possibility of identifying the optimal model in a subset of the population and testing it in a different validation cohort, as normally does in the machine learning/classification field. Moreover, a large number of predictors was initially included in the models, possibly leading to over-fitting problems that should be carefully considered when dealing with a limited number of subjects. Adding more data will allow to increase the power of the statistical analyses performed in this work and to further validate the promising findings about contralateral WM and GM changes suggesting the presence of plasticity processes.

## Conclusions

In conclusion, this work provided new evidence in favor of the suitability of dMRI-based microstructural indices for probing WM modifications and highlighted their potential as descriptors of microstructural feature changes in GM in ischemic stroke patients. To the best of our knowledge this is the first attempt of using 3D-SHORE-derived indices for studying microstructure in GM in both controls and patients, contributing a first step in bridging WM and GM diffusion signal modeling. In particular, the RTPP seems to be able to convey relevant information while being consistent across groups and time.

From the clinical point of view, our results provide additional evidence in favor of the hypothesis of the contralateral remodeling after stroke. The 3D-SHORE-derived indices performed as well as classical tensor-derived indices (FA and MD), achieving a high predictive power for clinical outcome over cortico-subcortical connections and a good discrimination between patients and controls at different time scales, further confirming their viability in ischemic stroke. Their combination can allow to convey a more detailed microstructural description, marking a step forward in the definition of a novel family of biomarkers. Finally, the detection of significant changes in GM across groups and in the patient longitudinal comparison provides a new perspective along the path of characterizing disease-related microstructural modulations which deserves further investigation.

## Author contributions

IBG and GM conceived and designed the experiments, interpreted the results of experiments and wrote the manuscript. IBG performed part of the statistical analyses. LB and SO analyzed the data and drafted the manuscript. MZ wrote the code for deriving all the indices, interpreted the results and drafted the manuscript. CG was involved in the design of the study and in data acquisition, interpreted the results of experiments, and drafted the manuscript.

### Conflict of interest statement

The authors declare that the research was conducted in the absence of any commercial or financial relationships that could be construed as a potential conflict of interest.
