# Supplementary material for: On the Viability of Diffusion MRI-Based Microstructural Biomarkers in Ischemic Stroke
Source: Front Neurosci. 2018 Feb 21;12:92. doi: 10.3389/fnins.2018.00092 (PMC5826355; doi:10.3389/fnins.2018.00092)
Supplement: Supplementary file 1 [file DataSheet1.docx]

***Supplementary Material***

**On the viability of diffusion MRI-based microstructural biomarkers in ischemic stroke**

**Ilaria Boscolo Galazzo*****, Lorenza Brusini, Silvia Obertino, Mauro Zucchelli, Cristina Granziera, Gloria Menegaz**

*** Correspondence:** Dr. Ilaria Boscolo Galazzo: ilaria.boscologalazzo@univr.it

**2. Supplementary Figures and Tables**

**2.1 Supplementary Tables**

**Supplementary Table 1. Patient demographics and clinical characteristics.** The following abbreviations are used: right middle cerebral artery (R MCA), left middle cerebral artery (L MCA) and left anterior cerebral artery (L ACA). Only the NIHSS-motor part is reported.

| **Patient** | **Gender** | **Age** | **Stroke Location** | **Arteries Involved** |  | **NIHSS Motor** | | |  | **Stroke Size**  **(# voxels)** |
| --- | --- | --- | --- | --- | --- | --- | --- | --- | --- | --- |
|  |  |  |  |  |  | ***tp1*** | ***tp2*** | ***tp3*** |  |  |
| 1 | F | 25 | L cortico-subcortical | L MCA |  | 13 | 4 | 3 |  | 8032 |
| 2 | M | 66 | L subcortical | L MCA |  | 5 | 2 | 2 |  | 1112 |
| 3 | F | 39 | R cortico-subcortical | R MCA |  | 7 | 4 | 3 |  | 76568 |
| 4 | F | 49 | R cortico-subcortical | R MCA |  | 8 | 3 | 1 |  | 15408 |
| 5 | M | 76 | L subcortical | L MCA |  | 6 | 2 | 2 |  | 7520 |
| 6 | M | 73 | L subcortical | L MCA  L ACA |  | 5 | 4 | 3 |  | 9400 |
| 7 | F | 67 | L cortico-subcortical | L MCA |  | 7 | 3 | 1 |  | 19336 |
| 8 | M | 62 | R cortico-subcortical | R MCA |  | 8 | 5 | 4 |  | 69832 |
| 9 | M | 35 | R cortico-subcortical | R MCA |  | 16 | 7 | 4 |  | 101360 |
| 10 | M | 69 | R cortico-subcortical | R MCA |  | 4 | 2 | 2 |  | 8752 |

**Supplementary Table 2. Grey matter regions.** List of the thirty-six regions and relative abbreviations considered for the region-based analyses.

| **ROI** | **Abbreviation** |
| --- | --- |
|  |  |
| Banks of superior temporal sulcus | bSTS |
| Caudal anterior cingulate cortex | cACC |
| Premotor area | PM |
| Cuneus | Cun |
| Entorhinal cortex | EC |
| Fusiform gyrus | FuG |
| Inferior parietal lobe | IPL |
| Inferior temporal gyrus | ITG |
| Isthmus cingulate gyrus | IGG |
| Lateral occipital cortex | LOC |
| Lateral orbito frontal cortex | lOFC |
| Lingual gyrus | LgG |
| Medial orbito frontal cortex | mOFC |
| Middle temporal gyrus | MTG |
| Parahippocampal gyrus | PHG |
| Supplementary motor area | SMA |
| Pars orbitalis | PORB |
| Pars triangularis | PTRI |
| Pericalcarine | PERI |
| Sensory cortex | SC |
| Posterior cingulate cortex | PCC |
| Primary motor area | M1 |
| Precuneus | PCN |
| Rostral anterior cingulate cortex | rACC |
| Rostral middle frontal gyrus | rMFG |
| Superior frontal gyrus | SFG |
| Superior parietal lobe | SPL |
| Superior temporal gyrus | STG |
| Supramarginal gyrus | SMG |
| Frontal pole | FP |
| Temporal pole | TP |
| Thalamus | Thal |
| Caudate | Cau |
| Putamen | Put |
| Hippocampus | Hipp |
| Amygdala | Amg |

**Supplementary Table 3. Reproducibility results for white matter.** Reproducibility for tract-based outcomes in terms of intra-class correlation coefficient (ICC) and intra-subject coefficient of variation (CV_intra_) for all the indices. CV_intra_ values are expressed as a percentage. A single representative value for each network was calculated. Part of this table was similarly described in (Brusini et al., 2016). CC = transcallosal circuit; CORT = cortical network; SUBCORT = subcortical network.

|  |  |  | **Tract-based** | | |
| --- | --- | --- | --- | --- | --- |
|  |  |  | **CC** | **CORT** | **SUBCORT** |
| GFA | ICC |  | 0.92 | 0.89 | 0.94 |
|  | CV_intra_ |  | 4.83 | 5.23 | 5.03 |
| PA | ICC |  | 0.90 | 0.82 | 0.96 |
|  | CV_intra_ |  | 3.98 | 5.23 | 4.71 |
| RTAP | ICC |  | 0.91 | 0.89 | 0.88 |
|  | CV_intra_ |  | 5.72 | 5.10 | 6.16 |
| RTPP | ICC |  | 0.90 | 0.59 | 0.88 |
|  | CV_intra_ |  | 2.83 | 4.92 | 3.10 |
| MSD | ICC |  | 0.83 | 0.67 | 0.95 |
|  | CV_intra_ |  | 2.06 | 3.40 | 1.13 |
| FA | ICC |  | 0.90 | 0.91 | 0.93 |
|  | CV_intra_ |  | 4.77 | 4.31 | 4.89 |
| MD | ICC |  | 0.85 | 0.89 | 0.94 |
|  | CV_intra_ |  | 7.74 | 3.73 | 3.50 |

**Supplementary Table 4. Model estimation results in the different linear regression models for clinical motor outcome prediction.** In each table the columns “coefficients”, “estimate”, “SE”, “*t*”, “*p*-value”, and “confidence interval” refer respectively to predictors survived to backward selection, corresponding numerical values, standard error, *t*-value, *p*-value, and *t*-test confidence interval. Tensor-based model, 3D-SHORE-based model and global microstructural model were evaluated for each of the three networks (Transcallosal [CC], Cortical [CORT] and Subcortical [SUBCORT]).

| **Coefficients** | **Estimate** | **SE** | ***t*** | ***p*-value** | **Confidence Interval** |
| --- | --- | --- | --- | --- | --- |
| *Stroke* | 2.03 e-5 | 0.7 e-5 | 3.103 | 0.015* | 1.33 e-5, 2.73 e-5 |

**Supplementary Table 4.1. The tensor-based prediction model for the CC network.** Statistically significance F(1, 8) = 9.626 with p = 0.015; RMSE: 0.596.

| **Coefficients** | **Estimate** | **SE** | ***t*** | ***p*-value** | **Confidence Interval** |
| --- | --- | --- | --- | --- | --- |
| MD | -10861.16 | 5727.24 | -1.896 | 0.100 | -16588.40, -5133.92 |
| *Stroke* | 2.21 e-5 | 0.6 e-5 | 3.837 | 0.006* | 1.33 e-5, 2.73 e-5 |

**Supplementary Table 4.2. The tensor-based prediction model for the CORT network.** Statistically significance F(2, 7) = 8.173 with p = 0.015; RMSE: 0.450.

| **Coefficients** | **Estimate** | **SE** | ***t*** | ***p*-value** | **Confidence Interval** |
| --- | --- | --- | --- | --- | --- |
| FA | 13.21 | 3.75 | 3.522 | 0.017* | 9.46, 16.96 |
| MD | -11061.26 | 3334.09 | -3.318 | 0.021* | 14395.35, 7727.17 |
| *Age* | -0.017 | 0.005 | -3.461 | 0.018* | -0.022, -0.012 |
| *Stroke* | 1.26 e-5 | 0.3 e-5 | 4.540 | 0.006* | 1.33 e-5, 2.73 e-5 |

**Supplementary Table 4.3. The tensor-based prediction model for the SUBCORT network.** Statistically significance F(4, 5) = 48.366 with p = 0.0004; RMSE: 0.053.

| **Coefficients** | **Estimate** | **SE** | ***t*** | ***p*-value** | **Confidence Interval** |
| --- | --- | --- | --- | --- | --- |
| RTPP | 0.231 | 0.089 | 2.577 | 0.033* | 0.142, 0.320 |

**Supplementary Table 4.4. The 3D-SHORE-based prediction model for the CC network.** Statistically significance F(1, 8) = 6.640 with p = 0.033; RMSE: 0.717.

| **Coefficients** | **Estimate** | **SE** | ***t*** | ***p*-value** | **Confidence Interval** |
| --- | --- | --- | --- | --- | --- |
| *Stroke* | 2.03 e-5 | 0.7 e-5 | 3.103 | 0.015* | 1.33 e-5, 2.73 e-5 |

**Supplementary Table 4.5. The 3D-SHORE-based prediction model for the CORT network.** Statistically significance F(1, 8) = 9.626 with p = 0.015; RMSE: 0.596.

| **Coefficients** | **Estimate** | **SE** | ***t*** | ***p*-value** | **Confidence Interval** |
| --- | --- | --- | --- | --- | --- |
| GFA | 115.57 | 6.34 | 18.220 | 0.003* | 109.23, 121.91 |
| PA | -159.02 | 10.82 | -14.700 | 0.005* | -169.84, -148.20 |
| MSD | -128516.13 | 6884.52 | -18.667 | 0.003* | -135400.65, -121631.61 |
| RTPP | -0.655 | 0.051 | -12.931 | 0.006* | -0.706, -0.604 |
| *Age* | -0.019 | 0.002 | -9.762 | 0.010* | -0.021, -0.017 |
| *Stroke* | 2.07 e-5 | 0.07 e-5 | 30.675 | 0.001* | 2.00 e-5, 2.14 e-5 |
| *NIHSS1* | -0.059 | 0.008 | -7.483 | 0.017* | -0.067, -0.051 |

**Supplementary Table 4.6. The 3D-SHORE-based prediction model for the SUBCORT network.** Statistically significance F(7, 2) = 777.113 with p = 0.001; RMSE: 0.002.

|  |  |  |  |  |  |
| --- | --- | --- | --- | --- | --- |
| **Coefficients** | **Estimate** | **SE** | ***t*** | ***p*-value** | **Confidence Interval** |
| RTPP | 0.524 | 0.136 | 3.861 | 0.006* | 0.388, 0.660 |
| MD | 14668.95 | 5833.88 | 2.514 | 0.040* | 8835, 20503 |

**Supplementary Table 4.7. The global microstructural prediction model for the CC network.** Statistically significance F(2, 7) = 8.690 with p = 0.013; RMSE: 0.431.

| **Coefficients** | **Estimate** | **SE** | ***t*** | ***p*-value** | **Confidence Interval** |
| --- | --- | --- | --- | --- | --- |
| GFA | -171.93 | 46.52 | -3.695 | 0.010* | -218.45, 125.41 |
| RTAP | 0.005 | 0.001 | 3.275 | 0.017* | 0.004, 0.006 |
| MD | -15848.52 | 7758.51 | -2.043 | 0.087 | -23607.03, -8090.01 |

**Supplementary Table 4.8. The global microstructural prediction model for the CORT network.** Statistically significance F(3, 6) = 5.243 with p = 0.041; RMSE: 0.483.

| **Coefficients** | **Estimate** | **SE** | ***t*** | ***p*-value** | **Confidence Interval** |
| --- | --- | --- | --- | --- | --- |
| RTPP | 0.539 | 0.117 | 4.624 | 0.002* | 0.422, 0.656 |

**Supplementary Table 4.9. The global microstructural prediction model for the SUBCORT network.** Statistically significance F(1, 8) = 21.379 with p = 0.002; RMSE: 0.357.

**Supplementary Table 5. ANOVA results (two-way ANOVA for repeated measures) for the longitudinal evaluation of grey matter outcomes in the patient group.** The two independent variables were Time Point (TP) and Region (ROI), while the dependent variable was the mean index value. The TP*ROI interaction is expressed in terms of F-ratio (degree of freedom, error) and p-values. * = significant values.

|  | **TP*ROI** | |
| --- | --- | --- |
|  | **F-ratio**  **(1,18)** | ***p*-value** |
| GFA | 1.611 | 0.002* |
| PA | 1.515 | 0.006* |
| RTAP | 1.161 | 0.184 |
| RTPP | 1.467 | 0.010* |
| MSD | 0.983 | 0.520 |
| FA | 1.918 | < 0.001* |
| MD | 1.756 | < 0.001* |

**2.2 Supplementary Figures**

**Supplementary Figure 1. Post-Hoc test results for the three-way mixed ANOVA (controls vs patients).** A) For each index, the first column represents the mean values for the controls while the second column the mean values for the patients, averaged across the first two time points. B) Post-hoc results expressed in terms of *p*-values for the significant interactions between Group and Region (ROI). Two different colormaps are used to display the *p*-values for the ROIs with significant differences between control and patient mean values (hot: Controls<Patients; cold: Controls>Patients). These values (p<0.05) are Bonferroni corrected for multiple comparisons.

**Supplementary Figure 2:** **Post-Hoc test results for the two-way ANOVA for repeated measures on patients.** A) For each index, the first column represents the mean values at *tp1*, the second column at *tp2* and the third at *tp3*. B) Post-hoc results expressed in terms of *p*-values for the significant interactions between Time Point (TP) and Region (ROI). Each column in the matrix refers to a specific statistical comparison between time scales, i.e. *tpi* vs *tpj* with *i = 1,2* and *j = 2,3* (first: *tp1 vs tp2*; second: *tp2 vs tp3*; third: *tp1 vs tp3*). Two different colormaps are used to display the *p*-values for the ROIs with significantly different values between the considered time scales (hot: positive difference, *tpi>tpj*; cold: negative difference, *tpi<tpj*). These values (p<0.05) are derived using Fisher's LSD (Least Significant Difference).
